# MicroRNAs as Diagnostic Biomarkers in Gastric Cancer

**DOI:** 10.3390/ijms131012544

**Published:** 2012-10-01

**Authors:** Ying Yin, Jun Li, Shujie Chen, Tianhua Zhou, Jianmin Si

**Affiliations:** Institute of Clinical Research of Gastroenterology, Sir Run Run Shaw Hospital, School of Medicine, Zhejiang University, 3 East Qingchun Road, Hangzhou 310016, China; E-Mails: dy1225@gmail.com (Y.Y.); lj_lcyxmajor@yahoo.cn (J.L.); chensj77@hotmail.com (S.C.); tzhou@zju.edu.cn (T.Z.)

**Keywords:** microRNAs, gastric cancer, biomarkers, diagnosis

## Abstract

Considering the high mortality rates and the unfavorable prognosis of gastric cancer (GC) as well as the lack of a clinical predictive marker, which is sufficiently sensitive to GC, it is of great significance to investigate new sensitive and specific markers for GC diagnosis. MicroRNAs (miRNAs) could be a practical form of potential biomarkers in the diagnosis of human disease, and they are confirmed to be closely associated with GC. In this review, we discuss the recent research results that indicate the feasibility and clinical applications of miRNAs in GC. Although several challenges remain to be addressed, miRNAs have the potential to be applied in the diagnosis of GC.

## 1. Introduction

Gastric cancer (GC) has a high incidence and ranks second in the leading causes of cancer mortality [1,2]. The prognosis of GC is highly dependent on the stage of the disease at diagnosis [3]. However, very few cases are detected at an early stage. Due to the high mortality rate and poor prognosis of the disease, primary prevention should be of high priority [4–6].

Recently, a cascade of premalignant conditions and lesions has been proposed, including chronic atrophic gastritis, intestinal metaplasia, and intraepithelial neoplasia (dysplasia) [7], whose identification and characterization might help to stratify the individual risk. The present classification systems of GC based on pathology has failed to efficiently predict the risk of sequential development from premalignant lesions such as intestinal metaplasia and even dysplasia to malignant lesions. Recent studies of molecular biology have revealed that certain gene alterations might be associated with some premalignant lesions. For example, a mutation in the p53 gene was detected in intestinal metaplasia lesions adjacent to GC [8], so was the over-expression of cyclooxygenase-2 [9]. Boussioutas *et al.* found a series of genes probably related with tumor progression by the expression profile of 124 gastric mucosa samples including gastritis, intestinal metaplasia, and adenocarcinoma [10].

As a result, it could be a long-term dynamic process from the premalignant conditions to GC, in which there could be the canceration of genes firstly, followed by malignant pathological changes in the gastric mucosa after the short-term malignant transformation progress [11–13]. And there could be a number of molecules associated with GC under research as possible prediction markers in the early stage of GC, even in late premalignant conditions. However, there is no good biomarker that is able to efficiently diagnose the corresponding stages of GC. To date, the existing cancer biomarkers such as MG7-Ag, CEA, CA199 and CA50 in clinical diagnosis cannot be effectively applied to the clinical diagnosis of GC because of their low sensitivity and specificity.

Besides the former gene biomarkers, microRNAs (miRNAs) could be another form of potential biomarkers in the diagnosis of human disease, and they are determined to be closely associated with cancers [14]. Meanwhile, it is promising that the miRNA network biomarkers could be applied to classify the expression data sets in different progression stages from premalignant conditions to GC.

## 2. The Mode of Action of MicroRNAs

MicroRNAs (miRNAs) are non-coding RNAs of approximately 19–25 nucleotides (nt), which suppress the translation of target genes by binding to their mRNAs. They are widely found in organisms such as plants, nematodes, fruit flies and mammals, and are conserved during evolution [15]. During miRNA biogenesis, genomic DNA is first transcribed by RNA polymerase II to produce the primary miRNA transcripts (pri-miRNA). The Drosha-DGCR8 complex initiates miRNA maturation by precise cleavage of the stem loop that is embedded in the primary transcript, producing a 60 to 70 nt miRNA precursor (pre-miRNA) in the nucleus [16]. Subsequently, the pre-miRNA is transported to the cytoplasm by means of GTP energy decomposition [17,18] and it is processed to a double-stranded RNA molecule of about 19 to 25 nt in length by the RNA endonuclease Dicer. Then, RNA interference is implemented through the action of the RNA-induced silencing complex (RISC) to regulate target genes. The final product is the single-stranded mature miRNA molecule, which can cleave the target mRNA when associated with RISC [19–21]. It is speculated that the total number of miRNAs may be much larger and that several of them have emerged only in primates.

## 3. Feasibility of Using MicroRNAs as Gastric Cancer Biomarkers

MicroRNAs have an important function in regulating RNA stability and gene expression by targeting mRNAs for cleavage or translational repression, and they are likely to influence the output of many protein-coding genes. The deregulation of miRNAs has been determined to correlate with cancer development and tumor progression [22]. They could play important roles in the regulation of almost all biological processes, including ontogeny, cell proliferation, apoptosis, migration, differentiation, metabolism and stress [23]. For example, miR-430 is involved in brain development in zebrafish [24]. Meanwhile, miR-181 could be involved in the regulation of B cell differentiation [25]. It is also found that miR-375 could regulate the development of mammalian islet cells and insulin secretion [26], while miR-221 and miR-222 could be involved in the regulation of angiogenesis [27]. In addition, genome-wide studies showed that miRNA genes are frequently located within regions of the loss of heterozygosity, amplification, fragile sites, and other cancer-associated genomic regions, suggesting the vital role of miRNAs in tumorigenesis [28].

Further investigation has shown that up-regulated and down-regulated miRNAs could be important in tumorigenesis as new broad-spectrum oncogenes and tumor suppressor genes in GC, respectively [29]. For example, findings demonstrated an oncogenic role for miR-372 in controlling cell growth, cell cycle and apoptosis through the down-regulation of a tumor suppressor gene, LATS2 [30]. Meanwhile, the over-expression of miR-650 may promote GC proliferation and growth of cancer cells, at least partially through directly targeting ING4 [31]. The down-regulation of mir-663 in tumor cells may contribute to the hyperplasia of aberrant cells, leading to the development of GC [32]. The studies showed that the aberrant over-expression of miR-126 and consequent SOX2 down-regulation might contribute to gastric carcinogenesis [33]. On the contrary, miR-9, miR-16 and miR-21 could target NF-kappaB1 and regulate the growth of GC cells, suggesting an impressive tumor suppressive activity in human GC pathogenesis [34,35]. Previous research provided a lot of important evidence that miR-148b targeted CCKBR and was significant in suppressing the growth of GC cells [36]. Furthermore, over-expression of let-7a resulted in a decrease in cell proliferation and G1 arrest, significantly suppressed anchorage-dependent growth *in vitro* and the tumorigenicity of GC cells in a nude mouse xenograft model [37].

Besides the association with tumorigenesis, further profiling studies have shown a correlation of miRNAs with proliferation, pathology, migration and invasion by means of targeting genes. The results suggested that miR-451 [38] and miR-141 could be involved in the development of GC through their inhibitory effect on cell proliferation [39]. Members of the miR-29 family could obviously inhibit cell proliferation, migration, and invasion of GC cells by targeting Cdc42 [40]. miR-544 could be a key regulator in switching cell cycles on or off in GC [41]. Meanwhile, the expression of miR-101 could be down-regulated in GC tissues and cells, and the ectopic expression of miR-101 significantly inhibits cellular proliferation, migration and invasion of GC cells by targeting EZH2, Cox-2, Mcl-1 and Fos [42]. Furthermore, miR-221 and miR-222 could regulate radio-sensitivity, cell growth and invasion of SGC7901 cells possibly via direct modulation of PTEN expression [43]. Jin *et al.* reported that both miR-192 and miR-215 were over-expressed *in vivo* and exerted cell growth and migration-promoting effects *in vitro* [44]. Liang *et al*. demonstrated that the over-expression of let-7f in GC could inhibit invasion and migration of GC cells through directly targeting the tumor metastasis-associated gene MYH9 [45]. Hayashi *et al.* showed that the cytotoxin-associated gene A of *Helicobacter pylori* could induce aberrant epigenetic silencing of let-7 expression, leading to Ras up-regulation [46]. Data indicated that miR-223 targeted FBXW7/hCdc4 expression at the post-transcriptional level and appeared to regulate apoptosis, proliferation, and invasion in GC [47]. In addition, miR-200b regulates ZEB2 expression and thus controls metastasis in GC [48]. miR-145 could suppress tumor metastasis by inhibiting N-cadherin protein translation, and then indirectly down-regulating its downstream effector MMP9 [49].

## 4. Clinical Application of miRNAs as Biomarkers in Gastric Cancer

A number of miRNAs are closely associated with GC, suggesting a potential role of these miRNAs in the diagnosis of GC [50]. By microarray technology, several miRNAs were demonstrated to be associated with GC with conspicuous expression changes. Some of these miRNAs were significantly up-regulated in GC endothelium compared to normal endothelium [51–55]. Meanwhile, other miRNAs such as mir-128b, mir-129 and mir-148 were reported to be down-regulated in undifferentiated GC tissue [53], which summarized in Table 1.

Presently, there have been some preliminary findings on the relationship between miRNA expression and GC development. Unique miRNAs were associated with the progression and prognosis of GC as meaningful prognostic markers [56,57], which is summarized in Table 1. Elevated miR-21 expression was significantly correlated with size and depth [58], and low expression levels of miR-451 [38] and miR-125a-5p was associated with enhanced malignant potential such as tumor size, tumor invasion, liver metastasis, and poor prognosis [59]. The patients with down-regulated miR-409-3p [60] and miR-221 [61] could be prone to suffer from lymph node metastasis. Furthermore, it was shown that miR-107 expression in GC tissues was an independent prognostic factor for overall survival (OS) and disease-free survival [62]. Additionally, multivariate analysis showed that the risk signatures could be independent predictors of OS, such as a seven-miRNA (miR-10b, miR-21, miR-223, miR-338, let-7a, miR-30a-5p, miR-126) [63] and a progression-related signature (miR-125b, miR-199a, and miR-100), which were associated with unfavorable outcomes in OS independent of clinical covariates including depth of invasion, lymph-node metastasis and stage [64]. Moreover, extracellular miR-196a detected in conditioned medium was strongly correlated with its cellular expression status, and the increased circulating miR-196a in patient serum was associated with GC disease status and relapse [65].

Generally speaking, the best cancer biomarkers for diagnosis should be peripheral blood markers in order to facilitate the health check-up of mass screening and follow-ups of those who are at higher risk. It is exciting that in 2008, three independent international research groups found the existence of stable miRNA in the peripheral blood of humans and other mammals almost at the same time. They revealed that the expression spectrum of particular peripheral blood miRNAs could constitute a “molecular fingerprint” for the diagnosis of some cancers and other diseases [66–68]. Within the past few years, it has become possible to detect the serum concentration of miRNA in GC [69–71]. There are only a few studies on the expression changes of miRNA in the serum of patients with GC, which are summarized in Table 2. Some miRNAs were reported to be up-regulated, including miR-20b, miR-20a, miR-17, miR-106a, miR-18a, miR-21 [72], miR-17-5p, miR-21, miR-106a and miR-106b [73]. Next-generation Solexa sequencing results demonstrated that 19 serum miRNAs were markedly up-regulated in GC patients compared to the controls, and a profile of five serum miRNAs (miR-1, miR-20a, miR-27a, miR-34 and miR-423-5p) was identified as a biomarker for GC detection. The analysis results showed that the serum miRNA-based biomarker could also indicate the progression stages of tumors [74]. Meanwhile, a study comparing pre- and post-operative plasma miRNA levels led to the identification of two miRNAs, miR-451 and miR-486, as potential biomarkers for GC, as they were highly abundant in plasma and showed a marked decrease in post-operative samples [75]. Tsujiura *et al.* reported that in these potential markers, the values of the area under the receiver-operating characteristic curve were 0.721 for the miR-106b assay and 0.879 for the miR-106a/let-7a ratio assay [73]. And miR-378 alone could yield a ROC curve area of 0.861 with 87.5% sensitivity and 70.73% specificity in discriminating GC patients from healthy controls [76]. The prognostic signature might be applicable and helpful for future treatment decisions. In addition, the latest study reported that the use of gastric juice miR-421 showed a remarkable improvement compared with the use of serum carcinoembryonic antigen alone [77].

Furthermore, there are still a lot of challenges in analyzing miRNAs, which concern quantification, normalization and data processing steps in miRNA analysis. The main quantification methods for analysis of miRNAs are the relative quantification by a stem-loop reverse transcription PCR [78], microarrays [79] and next-generation sequencing [69]. Quantitative RT-PCR has been widely used for the sensitive detection of low abundant circulating miRNAs with high reproducibility [78]. And sequencing technologies could result in a steep increase of the rate of newly described microRNAs [80]. Furthermore, due to the considerably variable results, it is needed for better standardization methods. So far, several normalization strategies for the analysis of circulating miRNAs are available especially housekeeping miRNAs, such as miR-16 [74] and RUN6B [73].

## 5. Conclusions

In conclusion, GC-specific miRNAs may improve future cancer diagnosis and prognosis due to the close association of miRNAs with GC in several stages. Many important functions of GC-specific miRNAs during the processes of cancer development were found and several promising miRNAs as biomarkers were reported. However, the quantification and normalization strategies should be standardized before any of the novel miRNA biomarkers is applicable for clinical routine, and the function of miRNAs needs to be further identified for the proper use of miRNA biomarkers in evidence-based medicine.

## Figures and Tables

**Table 1:** miRNAs in the tissue as diagnostic markers for gastric cancer.

Significance	MicroRNAs	Correlation with GC
Up-regulated	miR-21 [,8], miR-27a [49], mir-34b, mir-34c and mir-128a [50]	-
	miR-20b, miR-20a, miR-17, miR-106a, miR-18a, miR-106b, miR-18b, miR-421, miR-340 *, miR-19a and miR-658 [51], miR-223, miR-106b, miR-147, miR-34a, miR-130b *, miR-17, miR-98, miR-616 *, miR-181a-2 *, miR-185, miR-1259, miR-601, miR-196a *, miR-221 *, miR-302f, miR-340 *, miR-337-3p, miR-520c-3p, miR-575 and miR-138 [52], miR-181b [81]	

Down-regulated	mir-128b, mir-129 and mir-148 [50]	-

Correlated with prognosis	miR-21[53], miR-125b, miR-199a, miR-100 [59], miR-451 [38] and miR-125a-5p [54]	Correlated with tumor size and depth
	miR-451 [38] and miR-125a-5p [54]	Correlated with tumor invasion, liver metastasis
	miR-409-3p [55] and miR-221 [56], miR-125b, miR-199a, miR-100 [59] and miR-21 [82]	Correlated with lymph node metastasis
	miR-107 [57] miR-10b, miR-21, miR-223, miR-338, let-7a, miR-30a-5p, miR-126 [58], miR-125b, miR-199a, miR-100 [59], miR-185, miR-106b, miR-425, miR-106a, miR-20a, miR-21, miR-20b, miR-200a, miR-15a, miR-103, miR-107, miR-16, miR-34a [83] and miR-93 [84]	Correlated with OS
	miR-196a [60]	Correlated with cellular expression status

**Table 2:** Circulating miRNAs in the serum as diagnostic markers for gastric cancer.

References	Study design	Sample Size	Technology	Promising circulating miRNAs
Tsai *et al.* [60]	Different disease status and relapse	72 GC patients	Quantitative RT-PCR	miR-196a
Wang *et al.* [67]	Tumor *vs.* normal	30 GC patients *vs.* 39 normal controls	Quantitative RT-PCR	miR-21
Tsujiura *et al.* [68]	Tumor *vs.* normal	Screening: eight samples and associated tissues Validation: 69 GC patients *vs.* 30 healthy controls	Quantitative RT-PCR	miR-17-5p, miR-21, miR-106a, miR-106b and let-7a
Liu *et al.* [69]	Tumor *vs.* normal	Screening: 20 GC patients *vs.* 20 healthy controls Validation: 164 GC patients *vs.* 127 healthy controls	Solexa Sequencing Quantitative RT-PCR	miR-1, miR-20a, miR-27a, miR-34 and miR-423-5p Correlated with tumor stage
Konishi *et al.* [70]	Pre-operative *vs*. postoperative	Screening: three GC patients Validation: 56 GC patients *vs.* 30 healthy controls	Microarray analysis Quantitative RT-PCR	miR-451 and miR-486
Liu *et al.* [71]	Tumor *vs*. normal	Screening: seven GC patients *vs*. 10 healthy controls Validation: 40 GC patients *vs*. 41 healthy controls	Microarray analysis Quantitative RT-PCR	miR-378
Li *et al.* [85]	Tumor *vs.* normal	10 GC patients *vs*. 10 healthy controls	Quantitative RT-PCR	miR-223, miR-21 and miR-218
